# Hyperbaric Oxygenation in Pediatrics: Indications in The Light of Evidence –Based Medicine

**DOI:** 10.34763/devperiodmed.20192302.142148

**Published:** 2019-07-08

**Authors:** Jacek Siewiera, Judyta Mews, Katarzyna Królikowska, Bolesław Kalicki, Katarzyna Jobs

**Affiliations:** 1 Clinical Department of Hyperbaric Medicine at the Military Institute of Medicine, Warsaw, Poland; 2 Department of Pediatrics, Nephrology and Allergology, Military Institute of Medicine, Warsaw, Poland

**Keywords:** hyperbaric oxygen therapy, children, hyperbaric, chamber, HBOT indications, carbon poisoning, terapia hiperbaryczna, dzieci, komora hiperbaryczna, HBOT wskazania, zatrucie tlenkiem węgla

## Abstract

Hyperbaric oxygen therapy (HBOT), which is a centuries-old treatment, has now increasingly often been used in the pediatrie population. The basie indications for HBOT are well-known disease entities, i.e. carbon monoxide poisoning or decompression sickness. Due to the immunomodulatory properties of hyperbaric oxygen, attempts are made to use HBOT in the treatment of atopie dermatitis or inflammatory bowel diseases. The close cooperation between pediatricians and hyperbaric medicine teams is very important to obtain optimal results. The aim of this article is to present the mechanism of hyperbaric oxygen activity, and its influence on selected disease entities. The paper outlines new perspectives for HBOT in the pediatrie population.

## Streszczenie

Znana od wieków terapia tlenem hiperbarycznym (HBOT) od kilkudziesięciu lat ma coraz szersze zastosowanie w populacji pediatrycznej. Podstawowe wskazania to dobrze poznane jednostki chorobowe tj. zatrucie tlenkiem węgla czy choroba dekompresyjna. Ze względu na odkrycie immunomodulujących właściwości tlenu hieratycznego, coraz częściej podejmowane są do próby leczenia atopowego zapalenia skóry czy też chorób zapalnych jelit. Niezwykle istotna w uzyskiwaniu optymalnych efektów leczenia jest ścisła współpraca między pediatrami i zespołami medycyny hiperbarycznej. Celem pracy jest przybliżenie mechanizmu działania tlenu hiperbarycznego, jego wpływu na wybrane jednostki chorobowe oraz wskazanie nowych perspektyw dla hiperbarii w leczeniu dzieci.

## Introduction

Hyperbaric medicine is a discipline of science which originated from examining the physiopathology of diving. Although it has been developing from the eighteenth century, mainly with the advancement of underwater work technology, in 1959 it entered the world of modern medicine through the publication “Life without Blood” written by the Dutch cardiac surgeon, Boerema [1], who proved that the use of elevated atmospheric pressure makes it possible to sustain the life of living organisms completely erythrocyte–free, only due to plasma oxygen diffusion. The spectacular effect of his experiments lay at the basis of numerous publications whose subject was the use of hyperbaric oxygen therapy in clinical practice. Although his work is going to be 60 years old this year, hyperbaric oxygenation is still the subject of research in different areas of medicine, bringing forth both promising results and risks associated with its commercial use without clinical evidence.

**Photo 1:**
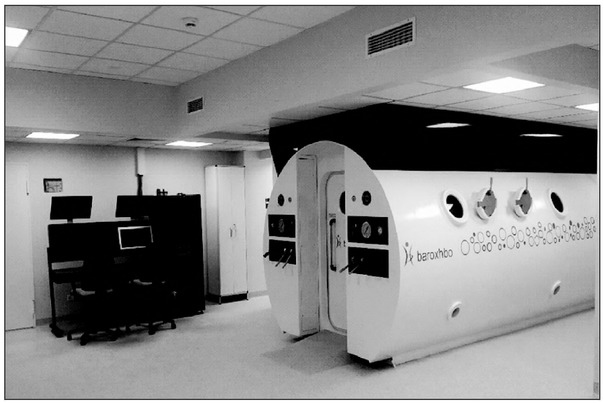
Hyperbaric chamber of the Clinical Department of Hyperbaric Medicine at the Military Institute of Medicine in Warsaw. Fot. 1. Komora hiperbaryczna Oddziału Klinicznego Medycyny Hiperbarycznej Wojskowego Instytutu Medycznego.

The aim of this paper is to present the current state of knowledge regarding the mechanisms of hyperbaric oxygen therapy (HBOT), its applications in pediatrics and, above all, indications established in the field of evidence-based medicine (Photo 1).

### Mechanism involved

**Photo 2:**
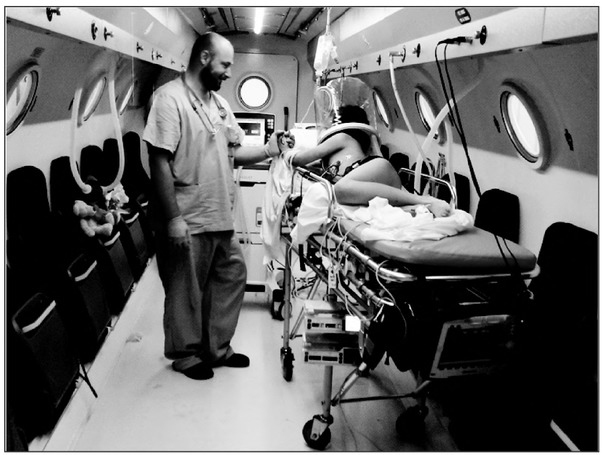
The use of an oxygen helmet in the intensive care unit as an effective treatment of an 8-year-old child with carbon monoxide poisoning. Fot. 2. Wykorzystanie hełmu tlenowego na stanowisku intensywnej terapii w skutecznym leczeniu 8-letniego dziecka zatrutego tlenkiem węgla.

Hyperbaric Oxygen Therapy (HBOT) is a method of treatment that involves using gas mixtures containing high oxygen concentrations under conditions of increased pressure. They are produced inside especially constructed steel rooms, called hyperbaric chambers, connected to systems which maintain overpressure. Due to specially built respiratory facilities, these chambers are used both for conscious patients and those who are mechanically ventilated in intensive care wards using a hyperbaric respirator and life support equipment. The treatment takes place at ambient conditions corresponding to those in the deep sea. By using appropriate mixtures of respiratory gases inside the hyperbaric chamber, it is possible to reach partial oxygen pressures significantly above those that are possible to obtain under atmospheric conditions. During planned treatment, the patient undergoes a series of healing sessions in a hyperbaric chamber (from one to several dozen), whose number and profile depend on the reasons for referring the patient for treatment (Photo 2).

Under conditions of standard (normobaric) atmospheric pressure the oxygen stream is calculated from the formula:

**I II III**

**SO2 = (HR x SV) x (Hgb x SaO2 x 1,34) + PaO2 x 0,003 Where**:

HR – heart rate

SV – stroke volume

Hgb – hemoglobin concentration in the blood

SaO2 – arterial oxygen saturation

PaO2 – partial pressure of oxygen in arterial blood

The first part corresponds to the minute capacity of the heart and the second describes the total amount of oxygen that in a given time unit can be transported in the blood with haemoglobin molecules. It is this part of the oxygen stream that is blocked by carboxyhemoglobin. The mechanism is similar to cases of severe anemia or haemorrhage. The third part of the formula describes the amount of oxygen transported in the plasma without being related to hemoglobin, depending on the partial O2 blood pressure. Under normobaric conditions, this part of oxygen is vestigial and constitutes a thousandth part of the arterial pressure of O^2^ in arterial blood, in accordance with the Bunsen coefficient of 0.003. Only in the face of a several-fold increase of oxygen partial pressure in the breathing mixture which can be achieved by raising the oxygen fraction and atmospheric pressure does the amount of molecules physically dissolved in the plasma increase sufficiently to maintain the life of an organism completely devoid of erythrocytes.

### Gas blockages and decompression sickness

Using the change in gas volume (described as the first mechanism of HBOT) is the treatment of choice in those diseases where hyperbaric therapy is a method of causal treatment, i. e.: decompression sickness and gas blockages [2, 3, 4]. Although these are rare conditions, whose occurrence in the group of patients under 18 is casuistic, nevertheless the frequency of their diagnosis in recent years has risen and this trend is likely to continue in technologically advanced societies. Because of the increasing popularity of underwater activity among children and of holiday air travel, the number of young patients suffering from decompression sickness is growing, because unfortunately, such adventures do not always go along with an increased awareness of the risks involved among the childrens caregivers. Meanwhile, many diving organizations follow the training of children from the age of 8.

**Photo 3:**
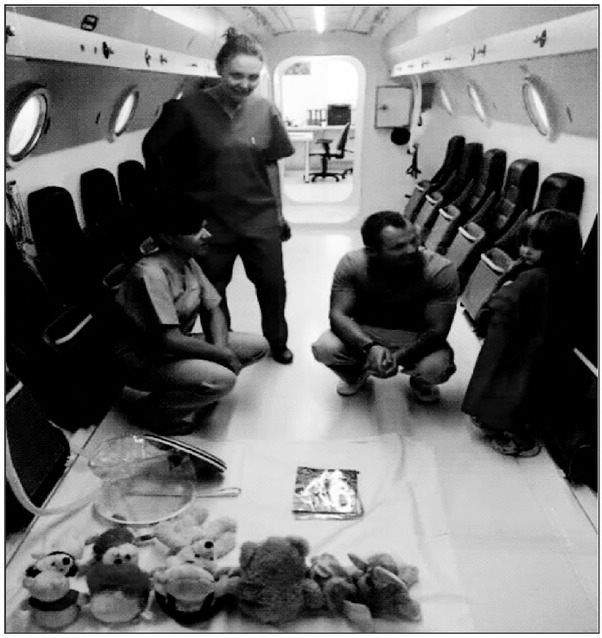
Preparation of the hyperbaric chamber for the treatment of a 4-year-old girl and her mother under the supervision of a physician of the Department of Clinical Hyperbaric Medicine. Fot. 3. Przygotowanie komory hiperbarycznej do leczenia 4-letniej dziewczynki oraz matki, pod nadzorem lekarza Oddziału Klinicznego Medycyny Hiperbarycznej.

As far as gas emboli are concerned, it should be emphasized that their occurrence is mainly post-traumatic or iatrogenic in nature, related to medical procedures requiring vascular access. A good example is the supply of intravenous contrast from an automatic syringe pump, or central vein cannulation [5, 6, 7]. These procedures are performed more and more often in the pediatric population, but complications of accidental vascular air infiltration may cause an immediate danger to life, particularly in those cases where there is a coexistence of a patent foramen ovale. In such circumstances, the only method of effective treatment is hyperbaric oxygenation, when according to the Boyle-Mariotte Law, the volume of intravascularly injected gas can be reduced in proportion to the pressure generated inside the hyperbaric chamber, until the free gas phase is completely dissolved in plasma [8]. The effectiveness of hyperbaric treatment depends on when it is applied and on the treatment profile specifying the time, pressure and type of breathing mix used [9]. (Photo 3).

### Acute carbon monoxide poisoning

CO (carbon monoxide) is produced by the incomplete combustion of materials that contain carbon [10]. CO poisoning, which is the most frequent cause of inhalation poisoning in the world [1], is not uncommon among children [10, 11]. Hyperbaric oxygen is the method of choice in CO poisoning in adults. Due to the consequences of carbon monoxide poisoning in children, so far there are no clear indications for this type of treatment in paediatrics [11]. The cells that are most exposed to the harmful effects of CO are those with the highest metabolic activity (cardiomyocytes and central nervous system cells). Clinical signs and symptoms of acute intoxications include headache, nausea and vomiting, dyspnea, vision abnormalities, muscular weakness, syncope, convulsions and coma. Children may present with general symptoms, such as nausea, vomiting, and diarrhea, and be mistakenly diagnosed as suffering from gastrointestinal disease. Small infants can present with poor feeding, irritability and vomiting. Many fire victims suffering from smoke inhalation and traumatic injuries may be in a coma and require mechanical ventilation. The history of exposure and the measurement of carboxyhemoglobin (COHb) levels can help to confirm diagnosis [12]. After exposure to the action of CO, the most important thing is to immediately leave the place where it occurred. Among adults, indications for initiating treatment with hyperbaric oxygen due to CO poisoning are a carboxyhemoglobin concentration over 16% or the occurrence of clinical symptoms. In case of the pediatric population, the criteria depend on the clinical symptoms observed. The concentration of carboxyhemoglobin in this case is only prognostic [11, 12] and the severity of the poisoning based on the COHb concentration cannot be estimated. Because of the lack of dependence between the concentration of carboxyhemoglobin and clinical symptoms, it is reasonable to treat each CO poisoning in a child as a life-threatening condition. Multicenter meta-analyses prove that hyperbaric oxygenation is better than normobaric treatment, because it prevents late neurological sequelae (i.e. headache, memory deficits, difficulty concentrating, sleep disorders) in patients poisoned with carbon monoxide [10, 11, 12]. It has been demonstrated that hyperbaric oxygen therapy in patients with severe CO poisoning has high efficacy and safety and results in a complete recovery after only one hyperbaric oxygenation exposure [10, 11, 12]. In people exposed to CO, oxygen therapy with 100% oxygen should be administered as soon as possible using an oxygen mask reservoir or a self-expanding bag. Nowadays the use of hyperbaric oxygenation is the recommended standard [11, 12]. The worse the patient’s clinical condition and the shorter the time that elapsed since the exposure, the more justified is this type of therapy. On the basis of current knowledge, children with symptoms of severe intoxication (IE. after loss of consciousness or neurological dysfunction, circulatory instability or haemodynamic disorders) should be immediately enrolled for treatment with hyperbaric oxygen therapy (HBOT) [10, 11, 12].

### Anaerobic and mixed bacterial infections of soft-tissues

Apart from diseases for which therapy in a hyperbaric chamber is a treatment method of choice, there is a range of indications with the proven effectiveness of HBOT as an adjuvant therapy. These include bacterial infections within the soft tissue involving both anaerobic and mixed flora. In the first case, the most common microorganisms are *Clostridium bacillus* that cause gas gangrene in surgical and post-traumatic wounds of various types [13]. The same situation concerns infections of the intestinal flora caused by *Clostridium perfringens* and *Clostridium difficile*, which can also cause symptoms of gas gangrene. Moreover, *Clostridium septicum*, in contrast to other microbes in this family, can cause symptoms of soft tissue infection, even though there is no wound in the patient’s body. These kinds of bacteria, like *Clostridium Difficile*, have the source in the intestinal mucosa and probably spread with the blood. All the pathogens mentioned above have the common characteristic of being in an absolutely anaerobic environment that allows the growth of bacterial colonies along with the massive production of exotoxins responsible for rhabdomyolysis and systemic signs of infection. The use of hyperbaric oxygenation that follows the increase of oxygen partial pressure in body tissues far above the physiological limits not only limits the proliferation of microorganisms and the production of exotoxins [14], but also has a bactericidal effect due to changing the pathogen environment [15].

In the case of mixed flora infections, the effectiveness of hyperbaric therapy is based on a slightly more complex mechanism of providing support for the non-specific immune system response. It is important both in the treatment of post-traumatic wounds with purulent exudation and edema of the wound area and abscesses which are a complication of the underlying disease. There are (so far only experimental) data that indicate the effectiveness of HBOT treatment of fistulas in the course of Leśniowski-Crohn’s disease, as well as in resistant bacterial infections caused by atopic dermatitis [16, 17]. One of the theories justifying the efficacy of oxygenation treatment in mixed bacterial flora infections claims that within the infected tissues there is a significant edema, which due to the increase of extravascular fluid volume leads to the closing of blood vessels at the microcirculation level. The lack of proper perfusion through arterioles and Zweifach’s loops results in a regional outbreak of tissue hypoxia around the source of infection [18, 19]. Oxygen concentration in this area can fall below physiological values, (i.e. 30 mmHg), which are essential for the organization of repair processes. The decrease in tissue oxygen concentration below 10 mmHg exceeds the threshold below which it is not possible to produce enough energy to maintain cell function, which results in the formation of local necrosis centers within the wound. Infection with necrotizing fasciitis is one of the most spectacular forms of this process. It can occur at a very early age [20, 21]. Meanwhile, an environment rich in oxygen is necessary for the proper performance of monocyte and neutrophil bactericidal function. In the phagocytosis process, post-activation cells consume about 50 times more oxygen in order to be able to produce free radicals, necessary to lyse the pathogen. This process, called a “respiratory burst”, cannot proceed properly in the case of oxygen deficiency in the vicinity of the macrophage. Local hypoxia caused by edema around the source of infection becomes a factor that prevents normal, unspecific immune system reaction. When using hyperbaric oxygenation, oxygen is delivered to the site of infection at a pressure many times higher than physiological (normobaric) conditions, omitting erythrocytes and a significant part of microcirculation, and providing oxygen molecules necessary for phagocytes to produce superoxide anions, followed by hydroxyl radicals and hypochlorites. The effectiveness of hyperbaric therapy in combination with antibiotics is based both on the direct germicidal properties of hyperbaric oxygen [22, 23, 24, 25] as well as on supporting the natural process of the non-specific immune response, decreasing both mortality and the necessity of surgical interventions [26, 27, 28].

### Extensive burns

Hyperbaric oxygenation has also proven its therapeutic efficacy as supportive treatment of severe burns classified as II on the three-point scale. Like in the cases described above, the presumed mechanism involved is based on correcting the oxygenation of tissues subjected to massive edema as a result of damage to the vascular endothelium. Moreover, burn patients need intensive fluid resuscitation to maintain the hemodynamic efficiency of the circulatory system [29, 30, 31]. When discussing burn disease with thermal damage to the second and third degree tissues (the burn zone around third degree burns with necrosis and focal points of necrosis is always classified as grade II), it is noteworthy that on subsequent days of hospitalization there is a radical increase of the risk for developing a widespread soft tissue infection which may lead to sepsis of bacterial origin [32, 33]. The mechanisms of the synergistic effect of antibiotics and hyperbaric oxygen described in the previous section also occurs in the course of burn disease, modifying the course of treatment of these patients in intensive care units [34, 35]. It should also be emphasized that the probable beneficial effect of hyperbaric oxygenation on patients in the phase of septic or hypovolemic shock has not been confirmed as effective in accordance with the European Baromedical Society (EBM) so far, although it finds a deep theoretical justification in the light of maintaining tissue oxygenation and vasoconstriction. Extensive, randomized controlled trials are necessary [36].

### Damage to the central nervous system

At the current state of medical science, one of the greatest hopes of hyperbaric medicine is associated with the impact of hyperbaric oxygenation on the central nervous system (CNS). After many years of preliminary research, groups of experts are interested in the use of this method of treatment in selected cases of the chronic consequences of stroke or hypoxemic lesions. It should be noted that in the light of the published studies, the justification for using hyperbaric oxygenation in patients with severe traumatic brain injury (sTBI) [37] is increasing, which may also apply to pediatric patients. In contrast to minor, chronic forms of TBI, where the treatment does not meet expectations [38], oxygenation in cases of severe CNS injuries results in the reduction of intracranial pressure and improvement on the Glasgow scale. Subsequent research, especially concerning the group of seriously ill patients requiring intensive care after CNS injuries, showed that it is necessary to iclude HBOT in the standard of care in acute TBI. When it comes to the dysfunction of the central nervous system, it is necessary to strongly emphasize the statement expressed by the European Baromedical Society (EBM) Consensus Conference on indications for treatment with hyperbaric oxygen, according to which autism or cerebral palsy are not indications for therapy with the use of HBOT. Regardless of the profitability of these procedures for the health care systems of different countries, it should be underlined that evidence-based medicine confirms the lack of effectiveness of this method in the treatment of autism and cerebral palsy [39].

## Summary

Clinical and basic science investigations have revealed that hyperbaric oxygenation has more benefits than normobaric treatment. Some of these include: restoration of normal oxygen gradients, enhancement of neutrophil function, suppression of *clostridil* toxin production, changes in systemic and cerebral vascular tone, reduction in tissue gas phase amount, prevention or amelioration of reperfusion injuries, and modification of the host inflammatory response. While all these mechanisms of hyperoxygenation apply to the entire age spectrum, there are some recently recognized biological mechanisms which have special relevance to pediatric patients. The administration of oxygen in hyperbaric chambers has been performed in many different ways. Many devices have been adapted to povide treatment to noncooperative infants.

In general, the indications for the use of hyperbaric oxygen in pediatric practice are very similar to those in adults. It needs to be stated that there are no specific scientific or professional committee recommendations concerning the treatment of the neonatal and pediatric population. The indications for hyperbaric oxygen therapy relevant to newborn infants and children, which have been approved by the Undersea and Hyperbaric Medical Society, are:

- Acute carbon monoxide poisoning,
- Cyanide poisoning,
- Arterial (cerebral) gas embolism,
- Compartment syndrome; acute traumatic peripheral ischemia,
- Clostridial mionecrosis (gas gangrene) and necrotizing soft-tissue infections,
- Compromised skin flaps and grafts,
- Chronic or refractory osteomyelitis,
- Osteoradionecrosis; radiation – induced soft – tissue injury,
- Intracranial abscess,
- Chronic, nonhealing wounds,
- Decompression sickness,
- Exceptional blood loss (anemia).

There is an urgent need to establish scientific and clinical bases for pediatric patients that allow the rational use and administration of HBOT.

Due to the high effectiveness and safety of this method of treatment, the number of indications for hyperbaric therapy is increasing. Currently attempts are being made to treat severe forms of atopic dermatitis or inflammatory bowel disease (Leśniowski-Crohns disease) in the pediatric population. It should be stated that the preliminary research results are promising.
